# Iatrogenic Microplastic Exposure: A Possible and Underrecognized Healthcare-Associated Exposure Framework in Human Medicotoxicological Risk

**DOI:** 10.3390/toxics14040302

**Published:** 2026-03-31

**Authors:** Hüseyin Çetin Ketenci, Hülya Kılıç

**Affiliations:** 1 Department of Forensic Medicine, Faculty of Medicine, Recep Tayyip Erdoğan University, 53100 Rize, Türkiye; hcetin.ketenci@erdogan.edu.tr; 2 Department of Medical Biochemistry, Faculty of Medicine, Recep Tayyip Erdoğan University, 53100 Rize, Türkiye

**Keywords:** intravenous contamination, dialysis fluids, healthcare-associated exposure, medical device safety, microplastic, nanoplastic, particle toxicology

## Abstract

Microplastics (MPs) are emerging environmental contaminants detected not only in water, soil, and air but also in human biological samples. To date, three main exposure routes have been identified. Currently, the principal exposure routes examined in scholarly works are oral, inhalational, and dermal. This paper explores iatrogenic microplastic exposure (IME) as an underrecognized healthcare-associated source of exposure and suggests that, in certain clinical contexts involving invasive, device-mediated, or direct systemic contact, IME may be considered a possible fourth route of exposure. IME is the introduction of microplastics into the human body through medical interventions. A literature-based conceptual review was conducted focusing on the materials and additives used in pharmaceutical formulations, intravenous systems, and medical devices. Particular attention was given to polymer-based excipients and plasticizers (e.g., phthalates, PEG, triacetin) found in enteric drug coatings and infusion packaging. Findings suggest that polymer-derived particles may enter systemic circulation via intravenous fluids, implantable devices, or oral medications, especially under conditions of heat, pressure, or prolonged contact. Such materials, though deemed biocompatible, may contribute to nanoplastic load and chronic exposure risks. Vulnerable groups such as neonates, oncology patients, and ICU populations may face disproportionate exposure. This calls for re-evaluation of plastic use in medical practice, improved regulatory oversight of pharmaceutical excipients, and innovation in plastic-free biomedical materials. Integrating this route into toxicological and epidemiological frameworks will enrich our understanding of microplastic-related health risks and broaden the scope of environmental health strategies.

## 1. Introduction

MPs are defined as plastic particles measuring less than 5 mm in diameter, which are increasingly recognised as a substantial source of contamination in environmental and biological systems. The presence of MPs has been reported in all ecosystem components, including soil, air, water, and the food chain, as a result of industrial activities, agricultural practices, and widespread plastic consumption [1]. In recent years, the detection of MP particles in various human biological samples (e.g., placenta, lung tissue, feces, and human blood) has revealed that these pollutants pose significant health risks beyond the environmental context alone. Despite the absence of definitive evidence regarding their impact on human health, the potential biological consequences of these substances, including the disruption of microbiota equilibrium, the induction of inflammatory responses, the generation of oxidative stress, and the induction of cellular toxicity, are subjects of mounting discourse within the preclinical and in vitro research domains [2]. In this context, the routes through which MPs enter the human body, the management of exposure, and the measures that can be taken in healthcare systems are of great importance. As emphasised in the contemporary scientific literature, MPs can enter the human body via three principal exposure routes: oral ingestion, inhalation, and dermal contact. Each of these routes represents a distinct interface between environmental sources of MPs and human physiology, collectively contributing to the systemic bioavailability and potential health effects of these ubiquitous pollutants [3]. This article provides a concise overview of the recognised exposure pathways of microplastics and their potential health implications, while also examining the concept of iatrogenic transfer within a scientific framework. In this context, IME is not intended to replace the established oral, inhalational, and dermal pathways, but rather to highlight healthcare-associated exposure scenarios that may overlap with them while also involving invasive, device-mediated, or direct systemic contact. Polymer-based materials, including those employed in drug packaging, intravenous fluids, medical devices, syringes, and catheters, have been demonstrated to elicit MP release even under conditions of sterilisation [4]. Based on the reviewed literature, IME may therefore be considered a possible fourth route of exposure in selected healthcare contexts, as well as an underrecognised conceptual framework for understanding medical contributions to microplastic burden.

## 2. Methodological Approach: Literature Search and Study Selection

This manuscript was designed as a literature-based conceptual review and was structured in accordance with the Preferred Reporting Items for Systematic Reviews and Meta-Analyses extension for Scoping Reviews (PRISMA-ScR). Given the heterogeneity of the available literature on healthcare-associated exposure to microplastics and nanoplastics in terms of study design, biological models, analytical methods, and reported endpoints, a scoping approach was considered more appropriate than a formal systematic review or meta-analysis.

**Figure 1:**
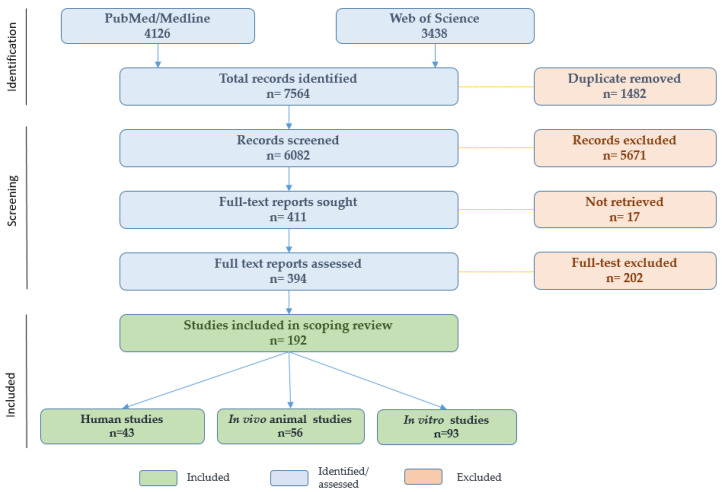
PRISMA-ScR flow diagram.

The literature search was conducted in June 2025 using PubMed/MEDLINE and Web of Science. The search strategy was developed to identify studies addressing human exposure to microplastics or nanoplastics, their biological distribution, toxicological effects, and their potential relevance to medical or healthcare-related exposure contexts. Search terms were constructed using combinations of controlled vocabulary and free-text keywords, including but not limited to “microplastic”, “nanoplastic”, “microplastics”, “nanoplastics”, “human”, “tissue”, “blood”, “placenta”, “semen”, “feces”, “thrombus”, “cell culture”, “in vitro”, “organoid”, “animal model”, “in vivo”, “toxicity”, “medical device”, “intravenous”, “dialysis”, “pharmaceutical packaging”, and “iatrogenic exposure”. The database search yielded 7564 records in total, including 4126 from PubMed/MEDLINE and 3438 from Web of Science. After removal of 1482 duplicate records, 6082 titles and abstracts were screened for relevance. A total of 411 articles were selected for full-text retrieval, of which 394 were assessed for eligibility. Ultimately, 192 studies were included in the scoping review, comprising 43 human studies, 56 in vivo animal studies, and 93 in vitro studies. The study selection process is summarised in the PRISMA-ScR flow diagram (Figure 1).

Studies were considered eligible if they were published in English and reported original data relevant to at least one of the following domains: (1) human studies demonstrating the detection, distribution, or clinical associations of microplastics/nanoplastics; (2) in vivo animal studies evaluating biodistribution, organ toxicity, mechanistic pathways, or systemic effects; and (3) in vitro studies investigating cellular uptake, cytotoxicity, oxidative stress, inflammatory signalling, barrier dysfunction, or other biological responses. The final evidence map was therefore organised into three principal categories: human studies, in vivo animal studies, and in vitro/cell culture studies. Studies were excluded if they were clearly unrelated to the biological or toxicological effects of microplastics/nanoplastics in humans or experimental models, focused exclusively on environmental occurrence without relevance to exposure biology, were conference abstracts lacking sufficient methodological detail, were duplicate publications, or did not have accessible full text. Review articles were screened for conceptual background and citation tracing; however, the main synthesis prioritised original research articles.

After database searching, titles and abstracts were screened for relevance, followed by full-text evaluation of potentially eligible studies. In addition, the reference lists of key articles were manually screened to identify further relevant publications. As the purpose of this review was to map the breadth of the available evidence and support the conceptual development of IME, no formal risk-of-bias assessment tool was applied. Instead, the included studies were synthesised narratively, with attention to model type, analytical method, exposure route, particle characteristics, and biological endpoints.

Overall, the literature was synthesised descriptively and comparatively to evaluate how evidence from human, animal, and cellular studies may support the plausibility of healthcare-associated microplastic exposure and its potential medicotoxicological implications. On the basis of this literature synthesis, we suggest that IME may be considered as a possible fourth route of exposure, particularly in healthcare settings involving invasive, device-mediated, or direct systemic contact.

## 3. General Characteristics and Effects of MPs

### 3.1. Overview of MPs

The term “MP” refers to synthetic polymer particles measuring less than 5 mm in size. These particles can be categorised as either primary microplastics, which are manufactured in these dimensions during the production process, or secondary microplastics, which are formed as a consequence of the physical, chemical, or biological degradation of larger plastic materials under environmental conditions [4]. The structural composition of these particles comprises polymers such as polyethylene (PE), polypropylene (PP), polyvinyl chloride (PVC), polystyrene (PS), and polyethylene terephthalate (PET). The particles exhibit high stability, a hydrophobic structure, and resistance to biological degradation. The chlorine compounds present in PVC contribute to its remarkable resistance to microbial degradation, contributing to its resistance to degradation and its potential to persist for prolonged periods under environmental conditions [5].

The accelerated production and consumption of plastic in recent decades has resulted in a series of irreversible problems in environmental and biological systems. It is estimated that approximately 9.2 billion tons of plastic were produced on a global scale between 1950 and 2017, with more than half of this amount being produced in the period subsequent to 2004. It is estimated that 400 million tons of plastic were produced in 2023 alone, and this amount is expected to reach 1.3 billion tons annually by 2060. While the increase in plastic production is driven by industrial and societal demand, inadequate waste management has played a major role in the environmental accumulation of discarded plastics and microplastics [6,7,8]. The result is that a significant portion of plastic waste is released into the environment without any control, causing persistent pollution in soil, water, and air. This phenomenon has precipitated a marked increase in the prevalence of MP, thereby propelling it to the forefront among environmental toxins. The extant literature on environmental science provides comprehensive documentation of the ecological effects of MP in aquatic systems [9,10,11,12]. However, the extent to which these substances affect soil ecosystems, and consequently agricultural production and human health, remains to be fully elucidated. It has been demonstrated that MP can diminish the water retention capacity of soil, exert a deleterious effect on photosynthesis and growth by being absorbed through plant roots, and lead to structural and functional damage to the soil microbiome [13,14,15,16,17]. Conversely, the presence of microbial biofilms on polymer surfaces has been shown to disrupt plant-microbe interactions, thereby reducing productivity and potentially leading to the release of toxic byproducts into the circulation [18,19]. In this regard, MPs are increasingly being recognised as a growing threat to biological systems.

### 3.2. The Impact of MPs In Vitro

Recent in vitro studies have examined the cytotoxic, genotoxic, inflammatory and oxidative effects that MPs can have on different cell types. These studies demonstrate that particle size, concentration, polymer type and surface modifications are critical factors in determining cellular responses. Experiments conducted on human epithelial, endothelial, immune and neural cell lines have produced results including decreased cell viability, increased reactive oxygen species (ROS), DNA damage, apoptosis and increased proinflammatory cytokine release. Common MP types such as PS, PE, and PVC have been used at the nanometre and micrometre scales. Notably, nanoplastics have been shown to penetrate the cell membrane, leading to lysosomal accumulation, mitochondrial dysfunction, and endoplasmic reticulum stress. While some studies have reported that MPs can cause mechanical damage to the cell membrane, others have shown that MPs are taken up into the cell via endocytosis, activating autophagy and inflammation-related pathways during this process. In terms of tissue-specific effects, different response profiles have been reported in intestinal, liver and lung cells. However, these findings cannot be directly generalised to human health due to the limited physiological representativeness of in vitro conditions. Cell culture environments do not fully reflect bioavailability, metabolism and immune interactions at the organism level. Across the reviewed in vitro studies, particle sizes, exposure concentrations, and incubation periods varied considerably depending on the experimental model, which should be taken into account when interpreting the reported cellular responses. Representative characteristics of selected in vitro studies, including particle size, exposure dose/concentration, and exposure duration, are summarised in Supplementary Table S1 [20,21,22,23,24,25,26,27,28,29,30,31,32,33,34,35,36,37,38,39,40,41].

### 3.3. The Effects of MPs In Vivo

In vivo studies conducted on rats, mice, zebrafish and other model organisms have demonstrated that exposure to MPs can result in pathophysiological changes to various organs and systems. The MPs used in these models are typically made of common polymers, such as PS, PE, PVC and PET and can be found in various sizes (nano-micro), shapes (spherical or fibrous), and concentrations. In animals exposed orally, increased intestinal permeability, villus damage, inflammation, microbiota imbalance, and oxidative stress are commonly reported findings. Additionally, microscopic accumulation, cellular dysfunction and changes in immune responses have been observed in organs such as the liver, kidneys, testes, brain and placenta. Systemic circulation and tissue accumulation of MPs can cause direct cellular toxicity and indirect effects on the endocrine, neurological, and reproductive systems. In the male reproductive system, decreased sperm quality, testicular damage, and hormonal changes have been reported. In females, hormonal irregularities and placental barrier disruption have been reported. Furthermore, exposure during pregnancy has been identified as having adverse effects on fetal development and neurological function. Oxidative stress, gliosis and behavioural changes observed in brain tissue indicate the neurotoxic effects of MP. However, the severity of these effects is determined by parameters such as exposure duration, dose, and particle characteristics [42,43,44,45,46,47,48,49,50,51,52,53,54]. Representative characteristics of selected in vivo studies are summarised in Supplementary Table S2.

### 3.4. The Effects of MPs on Human Health

In line with their widespread presence in the environment, there is increasing evidence of MPs in the human body. The main exposure routes include the oral, inhalation and dermal pathways. It has been demonstrated that these particles can enter the systemic circulation and reach tissues. Studies on MP detected in human placentae, lung tissue, faeces and blood, along with their associated biological effects, have yielded significant findings. Common plastic types such as PP, PS, and PET have been detected in placenta samples, indicating that the fetoplacental barrier can be breached. Furthermore, the presence of MPs in human blood was clearly demonstrated for the first time in 2022, confirming systemic distribution. Analysis of faecal samples revealed an average of over 20 MPs, with diet being identified as a significant factor influencing these results. MPs that accumulate in lung tissue via inhalation have been detected, particularly in surgical samples, raising concerns about environmental and occupational exposure. While the clinical effects are yet to be fully defined, it has been suggested that MPs may trigger cellular-level mechanisms such as inflammation, oxidative stress, and immune system activation. However, human studies are significantly limited by factors such as limited opportunities for direct biological sampling due to ethical constraints, small sample sizes, unstandardised particle identification techniques and contamination risks. Nevertheless, the current findings suggest that microplastics may have biological effects at the environmental, systemic, and organ levels. Therefore, further studies conducted on a larger scale, at multiple centres and using validated methods, are needed to improve our understanding of the effects on human health [55,56,57,58,59,60,61,62,63,64,65,66,67,68,69,70,71,72,73,74,75,76,77,78,79,80,81,82,83,84,85,86,87,88,89,90,91,92,93,94,95,96]. Representative characteristics of selected in vivo studies are summarised in Supplementary Table S3.

## 4. MPs Routes in the Human Body

### 4.1. Conventional Exposure Routes: Oral, Inhalation, and Dermal Routes

**Figure 2:**
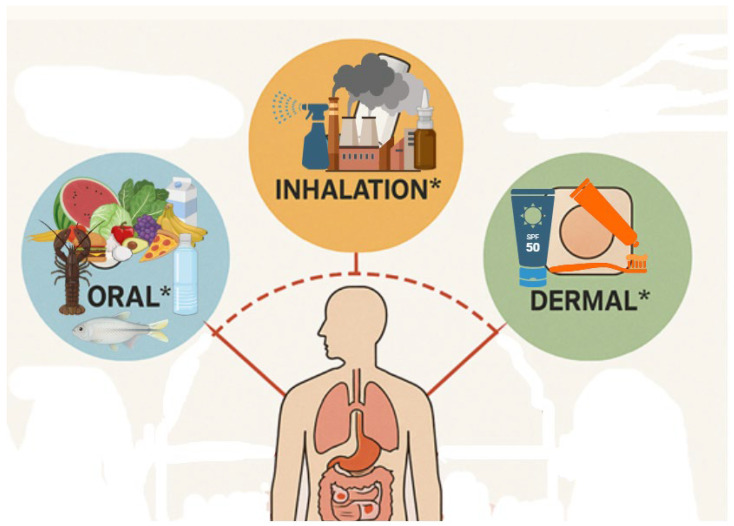
Conventional exposure routes of MPs. Created by the authors with the assistance of ChatGPT 4.5. based on author-provided content; not reproduced or adapted from any previously published source.

As demonstrated in Figure 2, MPs have the potential to enter the human body via three conventionally recognised exposure routes: namely, oral ingestion, inhalation, and dermal contact [3]. In this particular instance, it is widely accepted that oral ingestion represents the most significant route of entry. This occurs primarily through the consumption of contaminated foodstuffs and beverages, including seafood (especially filter feeders such as mussels and oysters), salt, bottled and tap water, milk, and even fruits and vegetables irrigated with polluted water or cultivated in plastic-contaminated soils. Once ingested, MPs have the potential to interact with the gastrointestinal (GI) tract. Research indicates that, depending on the size, surface chemistry and shape of the particles, some MPs have the capacity to traverse the intestinal epithelium via paracellular transport or endocytosis, thereby reaching the systemic circulation. The gastrointestinal route is also a key portal for additive chemicals leaching from MPs, which has the potential to lead to endocrine disruption or immune responses [97,98,99].

The inhalation route has emerged as an increasingly concerning pathway, particularly in urban, industrial, and indoor environments where airborne microplastic fibres and fragments are prevalent [100]. The sources of these materials include synthetic textiles, upholstery, packaging materials, and the degradation of plastic-based construction elements. These airborne MPs are capable of being inhaled and deposited along the respiratory tract, including the deep alveolar regions [101]. The capacity of MPs to circumvent mucosal barriers and persist within pulmonary structures is further highlighted by the detection of MPs in human lung tissue, bronchoalveolar lavage samples, and surgical biopsies. It is postulated that chronic respiratory exposure may result in local inflammation and oxidative stress, which may in turn contribute to fibrotic changes or respiratory disease [102,103,104,105,106,107,108,109,110,111,112].

The dermal route is regarded as being pertinent under specific circumstances, representing a notable consideration among the three. It has been demonstrated that intact human skin acts as an effective barrier against most microparticles. However, it is important to note that in cases of damaged, inflamed, or abraded skin, as well as in instances of long-term exposure, there may be a facilitation of limited transdermal penetration. Occupational exposure in industrial settings or the use of cosmetic products containing microbeads (such as exfoliants, toothpaste, or facial scrubs) increases the potential for contact-based absorption. Furthermore, the presence of plasticisers and chemical additives in direct-to-skin products has the potential to induce local or systemic effects via dermal absorption pathways [113,114,115,116,117,118,119,120,121].

Despite the fact that these conventional routes form the basis of current toxicological models, they may not fully encompass the diversity of exposure scenarios encountered in healthcare settings. The subsequent section therefore discusses iatrogenic microplastic exposure as an underrecognised healthcare-associated source of exposure that, in selected clinical contexts, may also be considered a possible fourth route.

### 4.2. Iatrogenic Microplastic Exposure as an Underrecognised Healthcare-Associated Source and Possible Fourth Route

Studies conducted to date have mainly emphasised oral, inhalational, and dermal pathways as the principal routes by which microplastics enter the human body. However, healthcare settings may also involve exposure scenarios that are not sufficiently highlighted within conventional environmental exposure models. In this context, the present article uses the term IME to describe an underrecognised healthcare-associated source of exposure and suggests that, in selected clinical circumstances involving invasive, device-mediated, or direct systemic contact, it may also be considered a possible fourth route of exposure. IME refers to the unintended transfer of microplastics or plastic-derived particles into the human body through medical procedures, pharmaceutical systems, and polymer-based healthcare materials. Many products commonly used in contemporary healthcare systems, including surgical gloves and masks, oxygen masks, intravenous fluid bags, syringes, catheters, infusion sets, probes, drains, medical packaging materials, surgical implants, pharmaceutical products, and contact-surface devices, are polymer-based [121,122,123,124,125,126]. Polymers such as PVC, PP, and PE, which are frequently used in the production of these items, may release microplastic and nanoplastic particles under certain conditions [127,128]. Processes such as sterilisation, exposure to elevated temperatures or ultraviolet light, improper storage, physical pressure, and mechanical friction may promote surface degradation and particle formation [129]. Under such conditions, particles may enter the bloodstream via intravenous systems or come into direct contact with body cavities through devices such as catheters. Exposure associated with medical materials may also occur through inhalational or dermal contact in selected contexts, for example, with masks and other polymer-containing supplies.

Evidence directly relevant to healthcare-associated exposure is beginning to emerge. One study detected microscopic particle contamination in intravenous fluid bags and infusion sets, with some particles shown to be plastic-derived [130]. Similarly, a systematic analysis of haemodialysis (HD) and peritoneal dialysis (PD) fluids identified suspicious plastic particles in all examined samples. FT-IR characterisation indicated that most particles were fibre-like and composed of common polymers such as PE, PVC, and ethylene-vinyl acetate (EVA). Although no significant difference in overall microplastic concentration was found between HD and PD solutions, larger particles were detected in HD fluids, and the estimated weekly total exposure of PD patients was approximately 50% higher than that of HD patients [131]. Taken together, these findings suggest that some medical products and treatment systems may warrant evaluation not only for microbiological contamination but also for possible particle-related contamination, particularly in vulnerable groups such as neonatal intensive care patients, individuals receiving long-term intravenous therapy, and immunosuppressed patients.

In addition to intravenous and dialysis-related systems, other iatrogenic contributors may include drug-packaging materials (e.g., blister packs and vial caps), oral dosage forms, and medical textile products [132,133]. A recent autopsy study detected 97 microplastic particles in stomach samples obtained from 26 cadavers. Fibres, fragments, and films were present in all cases, providing evidence that ingested microplastics can be retained in the stomach and may undergo transformation during digestion [134]. In the pharmaceutical context, enteric-coated systems are widely used to protect medications from gastric acidity and to facilitate targeted release within the gastrointestinal tract [135]. One important component of many of these systems is the use of plasticisers in polymeric coatings [136]. The addition of plasticisers may improve the mechanical properties and flexibility of the coating layer and help maintain dosage-form integrity until the intended dissolution pH is reached [137,138,139].

At the same time, it should be noted that plasticisers and polymeric excipients do not represent the same toxicological category as particle-based microplastics or nanoplastics. Nevertheless, they are relevant to the present discussion because some pharmaceutical systems may contribute more broadly to healthcare-associated exposure to plastic-derived materials. Commonly used plasticisers in coated formulations include triacetin (glyceryl triacetate), diethyl phthalate (DEP), dibutyl sebacate (DBS), polyethylene glycol (PEG), and triethyl citrate [140,141,142,143,144,145,146,147,148,149]. Among these, phthalate esters such as DEP and DMP have attracted particular toxicological concern because of their persistence in biological systems and their documented endocrine-disrupting potential [150,151,152]. In certain enteric-coated formulations, especially those involving methacrylate-based polymers, the generation of nano- or microscale particles during degradation or digestion has been discussed as a potential mechanism of exposure [153]. Preliminary findings have also suggested that some polymeric structures may disintegrate into smaller fractions during digestion, which may then cross the intestinal barrier [154,155,156]. However, the extent to which such processes contribute specifically to systemic microplastic burden remains insufficiently characterised and requires further investigation.

Taken together, these observations suggest that the healthcare system itself may represent a previously underappreciated context for plastic-related exposure. This perspective is not intended to replace established environmental exposure pathways, but rather to broaden current discussion by drawing attention to medical materials, procedures, and devices as possible contributors to cumulative particle burden in selected patient groups. At the same time, the available evidence remains limited by methodological heterogeneity, contamination concerns, and the scarcity of direct source-attribution studies in clinical settings [157].

At this juncture, a number of considerations may be articulated on global scales. The plastics employed in the fabrication of medical devices might necessitate reassessment regarding particle liberation and potential adverse effects, especially in circumstances involving recurrent or intensive exposure. Secondly, increased research and policy focus may be warranted for the advancement of safer alternative materials such as glass-based systems, biodegradable polymers, and other materials with reduced emissions. Subsequently, analyses of particle release from pharmaceutical packaging and intravenous fluid systems may be incorporated into comprehensive quality control protocols, complementing existing microbiological safety evaluations. Medical education, patient safety initiatives, and health policy development could integrate awareness of plastic-related exposure. Fifth, in high-risk clinical environments like intensive care, neonatal care, and oncology, the utilization of products with low particle emission may be advocated when practicable. A more thorough assessment of medical waste management protocols is warranted concerning secondary microplastic emission and ecological dispersion. Within this framework, the concept of IME can offer a valuable perspective for subsequent research in toxicology, epidemiology, and the biomedical sciences, additionally prompting a more comprehensive reassessment of biological safety within current healthcare practices.

## 5. Conclusions

This manuscript proposes IME as a clinically relevant exposure framework that may complement the conventional oral, inhalational, and dermal pathways described in the microplastics literature. The reviewed evidence suggests that plastic-containing medical devices, pharmaceutical packaging, intravenous systems, dialysis-related materials, implants, and other healthcare-associated products may contribute to unintended particle exposure, particularly in patients undergoing repeated or invasive interventions.

Although the extent of this exposure and its direct clinical consequences remain insufficiently characterised, the concept of IME provides a useful perspective for reconsidering the role of healthcare systems in human microplastic burden. The available literature supports the need for further analytical, toxicological, and clinical studies focusing on particle release from medical materials, exposure quantification in vulnerable patient groups, and the possible biological effects of repeated medical contact with plastic-based products.

Overall, integrating healthcare-associated exposure into microplastic research may help refine current toxicological models and support the development of safer material-use strategies in medicine. Broader regulatory and policy implications should be guided by stronger empirical evidence from future interdisciplinary studies.

From a practical perspective, several cautious considerations may be suggested:

Regulatory authorities may consider developing technical guidance for particle release from high-contact medical materials;

Manufacturers may be encouraged to evaluate and reduce particle-shedding potential under real-use conditions;

Healthcare systems may benefit from increased awareness in high-exposure settings such as intensive care, neonatal care, oncology, and dialysis;Future research should prioritize standardized analytical methods, source identification, exposure quantification, and clinical relevance.

## Data Availability

No new data were created or analyzed in this study. Data sharing is not applicable to this article.

## Supplementary Materials

The following supporting information can be downloaded at: https://www.mdpi.com/article/10.3390/toxics14040302/s1, Table S1. Summary of Selected In Vitro Studies on Microplastic/Nanoplastic Cellular Exposure. Table S2. Summary of Selected In Vivo Animal Model Studies on Microplastic/Nanoplastic Exposure. Table S3. Summary of Selected Human Studies on Microplastic/Nanoplastic Exposure and Detection.
