# Supplementary material for: Iatrogenic Microplastic Exposure: A Possible and Underrecognized Healthcare-Associated Exposure Framework in Human Medicotoxicological Risk
Source: Toxics. 2026 Mar 31;14(4):302. doi: 10.3390/toxics14040302 (PMC13119700; doi:10.3390/toxics14040302)
Supplement: Supplementary file 1 [file toxics-14-00302-s001.zip › toxics-4188950-supplementary.pdf]

Table S1. Summary of Selected In Vitro Studies on Microplastic/Nanoplastic Cellular Exposure

Reference	Cell Type / Model	Polymer	Particle Size	Dose / Concentration	Exposure Duration	Key Findings
Hu et al., 2025	Mouse fetal epidermal/dermal organospheres	PS	100 nm, 500 nm	NR	NR	100 nm particles penetrated to the center; 500 nm remained peripheral. Small MPs + UVA increased oxidative stress and apoptosis; elevated p53, Bax, SOD expression.
Choi et al., 2020	Human-derived cells (immune, fibroblast, cancer)	PS (irregular)	NR (irregular fragments)	Various	Acute	PS microfragments caused ~20× increase in acute inflammation in immune cells; ROS production and cell death in fibroblasts and cancer cells; membrane damage (LDH, hemoglobin release) correlated with surface roughness.
Choi et al., 2021	Human-derived cells (immune, RBC)	PE	Irregular particles	Various concentrations	Acute	Irregular/sharp PE particles caused higher cytotoxicity, IL-2, IL-6, TNF-α release, and hemolysis vs. smooth beads. Surface roughness and high concentration amplified toxicity.
Dong et al., 2020	BEAS-2B (human lung epithelial)	PS	NR	Low and high doses	Subacute	Low dose: cytotoxicity and inflammation. High dose: increased ROS, suppressed ZO proteins (barrier disruption), elevated IL-6, decreased α1-antitrypsin (COPD risk).
Goodman et al., 2021	A549 (human lung alveolar)	PS	1 µm, 10 µm	NR	72 h	Significant proliferation inhibition (viability >93%). 1 µm particles internalized; cytoskeletal morphological changes. First evidence of PS-MP-induced proliferation inhibition in human cells.
Han et al., 2020	Human-derived cells (immune)	PVC, ABS	Large and small (NR)	Various	NR	Large PVC particles induced IL-6 and TNF-α; small ABS at high concentration induced IL-6; large ABS increased TNF-α at all concentrations. Surface roughness quantified by curvature analysis.
Goodman et al., 2022	HEK 293, HepG2 (human kidney/liver)	PS	1 µm	Various	72 h	Proliferation inhibition in both cell lines (viability >94%). >70% internalization at 48 h. ROS increased at all doses/time points. Decreased GAPDH, SOD2, CAT gene expression → metabolic suppression and oxidative stress.
Hesler et al., 2019	Human intestinal and placental barrier models; embryotoxicity assay	PS-COOH	50 nm, 0.5 µm	NR	Acute	No acute toxicity or DNA damage. No significant barrier translocation, but intracellular accumulation observed. Mild embryotoxicity, no genotoxicity.
Hwang et al., 2019	PBMC, HMC-1 (mast cells), RBC (human)	PP (secondary)	20 µm; 25–200 µm	Various (DMSO solvent)	NR	Generally low cytotoxicity. Small particles + high concentrations elevated IL-6, TNF-α, histamine; potential hypersensitivity and local immune activation.
Wang et al., 2020a	Caco-2 (human colon)	PS	300 nm, 500 nm, 1 µm, 3 µm, 6 µm	Various	72 h	Uptake inversely correlated with size: 300 nm=73%, 500 nm=71%. Smaller particles adsorbed more BPA. MTT showed 300, 500 nm and 6 µm most cytotoxic. ROS↑, MMP↓. BPA-loaded particles more toxic.
Lehner et al., 2020	Caco-2 + HT29-MTX-E12 + macrophages/DCs (3D intestinal model)	Polyolefins, rubber particles	50–500 µm	823.5–1380.0 µg/cm²	6, 24, 48 h	No significant cytotoxicity, cytokine release (IL-8, TNF-α, IL-1β), or barrier disruption. Short-term exposure caused no measurable inflammatory effect.
Paul et al., 2023	Caco-2 + THP-1 macrophages + MUTZ-3 DCs (inflamed intestinal co-culture)	PLA, melamine formaldehyde, PMMA	Micro- and nano-scale	NR	NR	Model validated as sensitive tool for intestinal immune toxicity. Suitable for comparing normal vs. inflamed conditions.
Liu et al., 2020	Caco-2 (human colon)	PS	100 nm, 5 µm	NR	NR	In vitro digestion formed protein corona, reducing cytotoxicity and transport dysfunction but increasing pro-inflammatory cytokine response. 100 nm > 5 µm toxicity. Digestion alters toxicity profile.
Schirinzi et al., 2017	HeLa (epithelial), T98G (cerebral)	PE, PS + metal/carbon NMs	NR	10 ng/mL–10 µg/mL	24–48 h	No significant viability loss at low concentrations. Oxidative stress identified as cytotoxicity mechanism in both cell lines. Synergistic/antagonistic interactions with organic pollutants observed.
Stock et al., 2019	Caco-2, M-cell/goblet co-cultures, THP-1 macrophages	PS	1 µm, 4 µm, 10 µm	4.55×10^7 (1,4 µm); 1.49×10^6 (10 µm)	28 days (in vivo); NR (in vitro)	Limited particle uptake in vitro and in vivo. No tissue damage or inflammation. No disruption of THP-1 differentiation/activation. Oral PS-MPs did not cause acute health risks under experimental conditions.
Stock et al., 2021	Caco-2, HepG2, HepaRG (human intestine/liver)	PE, PP, PET, PVC	1–4 µm	Above realistic concentrations	NR	1–4 µm PE showed significantly higher intestinal translocation than PS. Cytotoxicity only at unrealistically high concentrations. Bioavailability depends on size and polymer type.

Reference	Cell Type / Model	Polymer	Particle Size	Dose / Concentration	Exposure Duration	Key Findings
Wang et al., 2020b	Caco-2 (human colon)	PS	300 nm, 500 nm, 1 μm, 3 μm, 6 μm	Various	72 h	Uptake rates: 73%, 71%, 49%, 43%, 30% by size. BPA adsorption higher on nano-sized particles. Nano-PS → high ROS; micron-PS → mitochondrial depolarization. BPA + nano-PS synergistically increased cytotoxicity.
Wu et al., 2019	Caco-2 (human colon)	PS	0.1 μm, 5 μm	≥20 mg/mL (0.1 μm); ≥80 mg/mL (5 μm)	NR	Both sizes: low toxicity on viability/oxidative stress/membrane. MMP disrupted by both (5 μm > 0.1 μm). Both inhibited ABC transporter activity; 0.1 μm as substrate competitor, 5 μm via ATP reduction. Increased arsenic toxicity.
Wu et al., 2020	Caco-2 (human colon)	PS microbeads	NR	12.5 mg/L, 50.0 mg/L	24 h	Dose-dependent viability reduction. RNA-seq: 442 differentially expressed genes (210↑, 232↓). NF-κB, MAPK, toll-like receptor pathways affected → intestinal inflammation and proliferation modulation.
Brown et al., 2001	Mono Mac 6 (human monocyte), A549 (lung epithelial)	PS	64 nm, 202 nm, 535 nm	NR	NR	64 nm particles caused significantly more neutrophil influx and LDH/protein release than larger particles in rat lungs. In vitro: only ultrafine PS increased Ca^{2+} and oxidant activity; IL-8 expression elevated in A549 only with ultrafine particles.
Palaniappan et al., 2022	L929 (murine fibroblast), MDCK (canine epithelial)	PS, PE	NR	1, 10, 20 μg/mL	6, 24 h	Dose-dependent decrease in cell viability. Metabolic activity increased per cell at higher doses. SOD3↑ (oxidative stress). TNF-α↑, IFN-β↓ → complex inflammatory dysregulation.
Brito et al., 2023	A549, HEK293, HeLa (human)	PS, PMMA	3 sizes per polymer	Multiple concentrations	NR	Uptake correlated with concentration (PS also with size 1.040 μm; PMMA peak at 400 nm). No significant viability/cycle changes (except positive controls). Intracellular thiol content and cytokine release significantly affected.
Yun, 2025	Human retinal microvascular endothelial cells, pericytes, astrocytes, microglia	PS	2 μm	NR	NR	PS reduced AKT/ERK1/2 activity → apoptosis in endothelial cells and pericytes. Inhibited tube formation, migration, proliferation; increased vascular permeability. Worsened retinopathy without promoting angiogenesis.
Huang et al., 2025	Human iPSC-derived cerebral organoids	PP nanoplastics	Nano-scale	NR	NR	Reduced organoid growth and neuronal differentiation; decreased TUJ1, MAP2, PAX6 expression.
Saraceni et al., 2025	Preadipocytes, hepatocytes, hypothalamic neurons, endothelial cells	PS-COOH, PS (plain)	5 μm	Wide range; toxic >100 μg/mL (COOH)	NR	Plain PS: no cytotoxicity across wide concentration range. COOH-PS: significant toxicity in neurons and endothelial cells at >100 μg/mL. Altered lipid accumulation in preadipocyte differentiation.
Zhang et al., 2025a	hiPSC-derived kidney organoids	PS	NR	0, 1.25–20 μg/mL (ref: 1.6 μg/mL blood level)	NR	PS-MPs increased mitochondrial oxidative stress, activated Bcl-2/Bax/caspase-9/-3 pathway → apoptosis, disrupting normal kidney organoid development.
Rahimi et al., 2025	HT-29 (human colon adenocarcinoma)	PS nanoplastics	Nano-scale	62.5–15.62 μg/mL	NR	PS-NPs caused dose-dependent cytotoxicity. L. plantarum mixture significantly reduced toxicity in 62.5–15.62 μg/mL range. Probiotics bound PS-NPs at 67–77% efficiency.
Tang et al., 2025	RD cells (human rhabdomyosarcoma)	PS	3 μm (MP), 100 nm (NP)	75, 150, 300 μg/mL	NR	PS-MPs/NPs internalized; reduced intracellular glucose uptake → insulin resistance. Mitochondrial ROS↑; MitoQ treatment improved mitochondrial function and IR.
Elbaghdady et al., 2025	BNL CL.2 (mouse hepatocytes)	PE nanoplastics	Nano-scale	IC_{50} = 334.9 μg/mL	NR	PE-NPs severely reduced viability (SRB assay). Biobran co-treatment preserved viability up to 85% even at highest PE-NP dose. PE-NPs caused G_2/M arrest and pre-G_0 cell death, reversed by Biobran.
Najahi et al., 2025a	Caco-2 (human intestinal)	PE, PET	1 μm, 2.6 μm	NR	72 h	Both MPs reduced viability and increased ROS (oxidative cytotoxicity). Apoptosis via Bax/Bcl-2↑ and caspase-3 activation. Autophagy induction (LC3-II↑, p62↓). Bafilomycin A1 confirmed autophagic flux.
Brouwer et al., 2025	hiPSC-derived intestinal epithelial cells	PET-TiO_2, PP-Talc, PVC, PA (true-to-life); PS (control)	True-to-life MPs	NR	NR	True-to-life MPs disrupted barrier integrity, increased ROS, stimulated cytokine release. PS (commonly used model) did not cause these effects. Protein corona composition correlated with in vitro response.
Zeng et al., 2025	Human peripheral blood neutrophils + AML12 hepatocytes (co-culture)	PS nanoplastics	Nano-scale	25 mg/kg (in vivo); NR (in vitro)	5 weeks (in vivo)	PS-NPs + glyphosate co-exposure amplified ROS, NETs formation, and pyroptosis via NLRP3 inflammasome. NLRP3 inhibition reduced NETs; NETs degradation reduced pyroptosis.
Michelini et al., 2025	Alveolar (A549/AT2) and bronchial (16HBE) epithelial cells	PS-Eu nanoplastics	Nano-scale	Non-acutely toxic doses	NR	No acute toxicity, but surfactant protein B↓ in alveolar cells; IL-6, TGF-β, ZO-1, MUC5B↑ in bronchial cells → pro-inflammatory response. Functional biomarkers important in toxicity assessment.

Reference	Cell Type / Model	Polymer	Particle Size	Dose / Concentration	Exposure Duration	Key Findings
Xiong et al., 2025	RAW264.7 (macrophage), MC3T3-E1 (osteoblast-like)	PS nanoplastics	Nano-scale	Up to 500 mg/L	NR	Macrophages: dose-dependent viability decrease and osteoclast differentiation↑. Osteoblast-like cells: no effect up to 500 mg/L. Suggests bone resorption promotion via macrophage pathway.
Edbauer et al., 2025	THP1-Blue™ NFκB monocytes	Micro- and nanoplastics	1 µm, 100 nm	NR	NR	Rapid internalization; inhibited E. coli phagocytosis and intracellular killing (concentration/time-dependent). No NFκB activation or IL-1β/IL-6 secretion. Endotoxin-enhanced phagocytosis was blunted at high plastic concentrations.
Lu et al., 2025	BV-2 microglia	PS nanoplastics	Nano-scale	25, 50, 75 µg/mL	NR	Reduced viability, G2 arrest, increased apoptosis and ROS. Elevated p65, TNF-α, IL-1β (gene + protein). NF-κB pathway activation → neuroinflammation.
Kang et al., 2025	AC16 human cardiac cells	PS nanoplastics	Nano-scale	NR	NR	PS-NPs activated TNF-α/NF-κB and P38/MAPK pathways in H9C2 myocardial cells (mouse in vivo). In vitro AC16 interventions confirmed FBP1 pathway role in PS-NP cardiac injury via gut-heart axis.
Mognetti et al., 2025	HepG2 (human liver)	PS nanoplastics	500 nm	NR	NR	Low direct toxicity, but PS-NPs increased ROS without affecting viability; reduced cadmium toxicity; inhibited fatty acid uptake. Indirect cellular effects through interaction with environmental pollutants.
Ma et al., 2025	hiPSC-derived cardiomyocytes (hiPSC-CM)	PS	1 µm, 0.05 µm	From 0.1 µg/L	Long-term, low dose	Dose/time-dependent viability reduction. Even 0.1 µg/L suppressed contractions and impaired calcium signaling. Hypertrophic CM: 0.05 µm worsened hypertrophy (cell size↑, proBNP↑). Mitochondrial dysfunction and ROS↑.
Zhang et al., 2025b	Trophoblast cells	PS (photo-aged vs. pristine)	NR	NR	NR	Photo-aged PS-MPs more cytotoxic than pristine form. Associated with physical property changes and increased lipid peroxidation. Environmentally aged MPs pose higher risk to placental health.
Morataya-Reyes et al., 2025	BEAS-2B (human bronchial epithelial)	PET nanoplastics + cigarette smoke condensate	NR	NR	Long-term (30 weeks)	Co-exposure significantly increased oxidative stress, DNA damage, cellular transformation, colony formation, and cell migration/invasion vs. single exposure. SLC7A11, NQO1, HSPA1A↑; LOX, FN1↓.
Cheng et al., 2025a	Human trophoblast cells	Micro- and nanoplastics	NR	NR	NR	MNP exposure disrupted syncytialization; decreased syncytialization markers. PERK/eIF2α/ATF4 pathway activated in both mouse placental tissue and human trophoblasts; inhibition partially reversed the defect.
Chen et al., 2025	SH-SY5Y (human neuronal)	PS nanoplastics	Nano-scale	0.75 mg/mL	NR	Reduced cell viability; TTM (copper chelator), NAC, and PD98059 (MAPK inhibitor) mitigated effects. PS-NPs activated ERK/MAPK via oxidative stress → neuronal cuproptosis and cognitive impairment.
Merlo et al., 2025	Bovine oocytes (in vitro maturation)	PS nanoparticles	100 nm, 30 nm	100–200 µg/mL (100 nm); 70 µg/mL (30 nm)	In vitro maturation period	High doses reduced nuclear and cytoplasmic maturation; increased oocyte degeneration. ROS↑, GSH↓. Dose-dependent internalization in oocyte and cumulus cells.
Park et al., 2025	Human cerebral organoids; mouse brain	PS/microplastics	50 nm, 100 nm	NR	NR	50 nm particles penetrated deeper than 100 nm. Significant viability reduction and expression changes in neurotoxicity-related genes in organoids.
van Boxel et al., 2025	BeWo b30 + HUVEC (Transwell placental model)	PS	50 nm, 200 nm, 1000 nm	1–10 µg/mL	72 h	50 nm: 8.7% translocation; 200 nm: 1.2%; 1000 nm: not detected. Both layers internalized 50/200 nm particles. No mitochondrial, oxidative stress, or gene expression changes at tested doses. 17-α-estradiol ↓17% at 1 µg/mL 50 nm.
Cui et al., 2025	Porcine oocytes (in vitro maturation)	Microplastics (NR polymer)	NR	NR	NR	MP exposure caused polar body extrusion failure, spindle/chromosome abnormalities, cortical actin disruption. Reduced choline and creatine metabolism; mitochondrial dysfunction; GSH↓; DNA damage and apoptosis.
Gutiérrez-García et al., 2025	BEAS-2B (human bronchial epithelial)	PET nanoplastics	Nano-scale	NR	30 weeks (chronic)	No genotoxicity at 24 h or 15 weeks. At 30 weeks: DNA damage, anchorage-independent growth, and invasive potential increased. Oncogene expression↑; progressive transcriptomic changes associated with lung cancer pathways.
Simpson et al., 2025	Keratinocytes, fibroblasts (human skin)	Nanoplastics with environmental corona	Nano-scale	NR	NR	Environmental corona altered cell entry pathways and intracellular localization. In keratinocytes: TLR4, TNF-α, COX-2, IL-8↑ → inflammatory and chemotactic responses.
Yang et al., 2025	HUVECs (human umbilical vein endothelial cells)	PS	5 µm	1 µg/mL, 10 µg/mL	NR	Oxidative stress↑, BMP pathway activation, EndMT phenotypic changes (E-cadherin↓, N-cadherin↑). Linked to atherosclerosis plaque progression.

Reference	Cell Type / Model	Polymer	Particle Size	Dose / Concentration	Exposure Duration	Key Findings
Zou et al., 2025	Caco-2 (human intestinal)	PET MPs + PFOA	NR	NR	NR	PFOA caused oxidative stress, mitochondrial dysfunction, and cytotoxicity; inhibited ZO-1. PET MPs increased membrane impermeability → enhanced PFOA accumulation → synergistic barrier disruption.
Liu et al., 2025	Cardiomyocytes (in vitro); mouse heart (in vivo)	PVC nanoplastics	Nano-scale	Dose-dependent	NR	PVC-NPs accumulated in lysosomes and mitochondria; disrupted autophagosome-lysosome fusion → autophagic flux impairment; reduced MMP and ATP; decreased TCA cycle activity → cardiac dysfunction.
Najahi et al., 2025b	HepG2 (human hepatoma)	PET, PE	NR	10 µg/mL	72 h	Viability paradoxically increased, but ROS↑ and mitochondrial dysfunction. MtDNA integrity and MMP↓. Autophagy activated (LC3↑, p62↑); autolytic process blocked by bafilomycin.
Gu et al., 2025	RLE-6TN (rat alveolar type II epithelial)	PS	NR	0, 10, 100, 500 µg/mL	48 h	PS-MPs disrupted TCA cycle, inhibited oxidative phosphorylation, caused mitochondrial dysfunction → excessive mitophagy. Potential amplification of inflammatory responses.
Cheng et al., 2025b	Human iPSC-derived liver organoids (LOs); mouse liver (in vivo)	PP (pristine), PP (UV-aged)	Mean 7.60 µm (PP); 6.91 µm (aPP)	75 ng/mL	NR	Transcriptomics: NADH dehydrogenase and ATP synthesis affected (especially aged PP). Metabolomics: cysteine/methionine metabolism altered. Homocysteine identified as potential systemic biomarker.
Kim et al., 2025	Brain (global cerebral ischemia model, mouse); in vitro	Microplastics	0.5 µm	50 mg/kg (oral)	NR	MPs accumulated in brain post-ischemia; increased neuronal death, myelin/microtubule damage, neuroinflammation. Elevated IL-6, TNF-α, phospho-tau (S396). Potential link to neurodegeneration (Alzheimer-like).
Marcellus et al., 2025	Caco-2 (mono, differentiated mono, tri-culture Transwell)	PS	50 nm, 500 nm	NR	NR	Only 50 nm and 500 nm internalized in undifferentiated cells; no toxicity at tested doses. No barrier disruption. 50 nm showed limited translocation to basolateral side vs. tri-culture (with mucus layer).
Brynzak-Schreiber et al., 2024	HT29, HCT116, SW480, SW620 (colorectal cancer)	PS	0.25 µm, 1 µm, 10 µm	Various	NR	Uptake in all lines (highest HCT116); particles distributed to daughter cells without elimination. 0.25 µm exposure significantly increased cell migration. Accumulation in 2D and 3D cultures; no effect on proliferation.
Shen et al., 2022	HL7702 (human hepatocyte)	PS microplastics	0.1 µm, 1 µm	1 mg/L (fluorescent)	24 h	Particles accumulated intracellularly. Mitochondrial and nuclear DNA damage; cGAS/STING pathway activation; NFκB translocation → hepatic fibrosis pathway.
Pontecorvi et al., 2023	Human vaginal keratinocytes	PE	200 nm–9 µm	High concentrations	Acute + chronic	High conc: intracellular accumulation, reduced viability, apoptosis↑, morphological changes, junction/adhesion protein disruption, actin disorganization. Altered miRNA expression (oxidative stress, barrier function). Persistent epigenetic changes (DNMT, demethylase) suggesting accelerated aging/malignant transformation risk.
Wang et al., 2025a	Corneal and conjunctival epithelial cells	PE	NR	Dose-dependent	NR	PE exposure dose-dependently reduced viability and triggered apoptosis in ocular surface epithelial cells.
Remigante et al., 2024	Human erythrocytes	PS-NP, PS-MP	Nano and micro scale	Various	NR	Abnormal cell shapes (acanthocytes, echinocytes, leptocytes)↑; ROS, lipid peroxidation, protein oxidation↑. Band 3 transporter dysfunction; oxidized hemoglobin↑; membrane protein clustering. Impaired systemic homeostasis.
La Maestra et al., 2024	THP-1 monocytes	PS (UVB-aged, 1 µm and 5 µm)	1 µm, 5 µm	NR	NR	UV-aged MPs increased intracellular ROS and MDA (lipid peroxidation). Genotoxic damage more pronounced with smaller and UV-aged MPs. Environmental degradation enhances toxicity.
Persiani et al., 2025	Human vascular smooth muscle cells (VSMC)	PE, PS (virgin and photo-degraded)	NR	NR	NR	Both MPs reduced viability, triggered apoptosis, activated inflammation (RUNX-2, galectin-3, inflammasome complex). Photo-degraded MPs maintained or amplified effects. Potential link to atherosclerosis and vascular calcification.
Yin et al., 2024	CAR-T cells (CD19 and BCMA targeted)	Microplastics (unspecified)	100 nm, 9 µm	Various	NR	MPs significantly increased apoptosis, ferroptosis, and T cell exhaustion in CAR-T cells via mTORC1 hyperactivation. Rapamycin partially reversed cellular damage.
Kim and Lee, 2024	Mouse cerebrocortical neurons	Nanoplastics	100 nm	Various	24 h	Dose-dependent neuronal accumulation; increased dopamine D1 and D2 receptor levels and co-expression rate. No changes in dopamine synthesis, reuptake proteins, or structural markers. Potential link to psychiatric dopamine-related disorders.

Reference	Cell Type / Model	Polymer	Particle Size	Dose / Concentration	Exposure Duration	Key Findings
Schnee et al., 2024	Human breast epithelial and breast cancer cells	PS	Nano and micro scale	Various	NR	Cell-type and size/dose-dependent uptake (highest MDA-MB-231-DSP1-7; lowest M13SV1). No effect on colony formation or cell fusion. Slight, partially significant stimulation of proliferation and migration in some lines.
Manabe et al., 2024	RAW264.7 (macrophage), THP-1 (monocyte)	PE (UV-degraded vs. pristine)	NR	NR	24 h	Only UV-degraded PE triggered programmed cell death (no caspase-3 activation). Lysosomal acidic content reduced; p62 accumulation; autophagosome↑ → autophagic flux disruption → cell death.
Hong et al., 2024	Renal tubular epithelial cells	PS microplastics	NR	NR	Chronic	Ferroptosis induction; TGF-β1 release; fibroblast activation → renal fibrosis pathway identified.
Wang et al., 2021	HK-2 (human proximal tubular)	PS	2 μm	NR	NR	PS-MP uptake → mitochondrial ROS↑, Bad protein↑, ER stress, inflammation, autophagy (LC3, Beclin-1)↑; MitoTEMPO alleviated effects. MAPK and AKT/mTOR phosphorylation changes.
Chen et al., 2022	HEK293 (human kidney)	PS microplastics	~3.54 μm	3 ng/mL and 300 ng/mL (realistic concentrations)	NR	Membrane adhesion and internalization. Both doses induced apoptosis (MMP depolarization) and autophagy (autophagosome formation). ROS↑; HO-1-mediated antioxidant suppression. Low dose: 33 cytokines↑; High dose: NLRP3↓ (autophagic buppression), 35 cytokines↑, ZO-2 and α1-AT loss → barrier disruption, acute kidney injury risk.
Sun et al., 2023	HEK293 + mouse kidney (C57BL/6)	PS + DEHP (combined exposure)	5 μm	Various	NR	PS+DEHP co-exposure: strongest ROS induction. AMPK/ULK1 and Ppargc1α/Mfn2 mRNA/protein↑; LC3/Beclin-1↑; autophagosome accumulation confirmed. DEHP+PS synergistically activated AMPK/ULK1 axis → autophagy and nephrotoxicity.

Abbreviations: PS = polystyrene; PE = polyethylene; PP = polypropylene; PET = polyethylene terephthalate; PVC = polyvinyl chloride; PMMA = polymethylmethacrylate; PLA = polylactic acid; PA = polyamide; NR = not reported; MP = microplastic; NP = nanoplastic; ROS = reactive oxygen species; MMP = mitochondrial membrane potential; LDH = lactate dehydrogenase; MDA = malondialdehyde; IL = interleukin; TNF-α = tumor necrosis factor alpha; NF-κB = nuclear factor kappa B; SOD = superoxide dismutase; GSH = glutathione; MAPK = mitogen-activated protein kinase; ABC = ATP-binding cassette; ZO = zonula occludens; hiPSC = human induced pluripotent stem cell; PBMC = peripheral blood mononuclear cells; HUVEC = human umbilical vein endothelial cell; DC = dendritic cell.

Table S2. Summary of Selected In Vivo Animal Model Studies on Microplastic/Nanoplastic Exposure

Reference	Animal Model / Species	Polymer	Particle Size	Dose	Route of Exposure	Duratio n	Key Findings
Huang et al., 2025	Pregnant mice + offspring; human iPSC cerebral organoids	PP nanoplastics	Nano-scale	NR	NR	Gestation al	Fetal cortex: impaired neuronal differentiation and proliferation. Offspring: spatial memory deficits, reduced motor coordination, increased anxiety-like behavior.
Wen et al., 2025	Male mice (oral gavage)	PS nanoplastics + Mn	0.1 μm	NR	Oral gavage	NR	Co-exposure: synergistic increase in testicular tissue damage, sperm abnormalities, hormone dysregulation. Increased apoptosis, oxidative stress, and impaired spermatogenesis. Baicalin/YTHDC2 mechanism identified.
Zhou et al., 2025	Male C57BL/6 mice	PS-MPs + DEHP	NR	PS: 20 mg/kg; DEHP: 200 mg/kg	Oral	28 days	Co-exposure: severe seminiferous tubule disorganization. PPARγ signaling pathway central to testicular damage. Combined exposure more harmful than individual exposures; lipid metabolism disruption.
Gu et al., 2025	Shaoxing ducklings	PS microplastics	NR	1 mg/L, 100 mg/L	Oral	4 weeks	Pulmonary histopathological changes, inflammatory cell infiltration, TLR4/LPS pathway activation. TCA cycle disruption, oxidative phosphorylation inhibition, mitochondrial dysfunction → excessive mitophagy.
Mans et al., 2025	Adult zebrafish	PS nanoplastics	44 nm	Nanoplastic-enriched diet	Diet	14 days	Increased microglial immunoreactivity (4C4) in telencephalon. No change in GFAP (astrocyte marker). Short-term NP exposure activates microglial response without astrogliosis.
Hsu et al., 2025	Mice	PS nanoplastics	Nano-scale	NR	NR	NR	NPs altered miR-501-3p and miR-700-5p → reduced ZO-1 and MUC-13 expression → increased intestinal permeability. Gut dysbiosis (Ruminococcaceae↑, Lachnospiraceae-related MUC-13

Reference	Animal Model / Species	Polymer	Particle Size	Dose	Route of Exposure	Duration	Key Findings
							suppression). Extracellular vesicle-delivered miRNAs as mechanism.
Xiong et al., 2025	Pregnant/lactating mice + offspring	PS nanoplastics	100 nm	10 mg/L (low), 100 mg/L (high)	Drinking water	Gestational + lactation	Low dose: ↑osteoblast count, bone mineral density and content. High dose: ↓femur growth plate thickness. Both doses: skeletal gene expression/metabolic dysregulation; NET suppression with abnormal immune infiltration at high dose.
Lu et al., 2025	Mice	PS nanoplastics	~100 nm	10, 20, 50 mg/kg	NR	NR	Brain PS-NP accumulation → anxiety-like behavior and cognitive impairment. Prefrontal cortex tissue damage, Iba1 and GFAP↑. Transcriptomics: Pbx3, Ecell, Crb1, Ngb genes; NIK/NF-κB inflammatory pathway activated.
Kang et al., 2025	Mice	PS nanoplastics	80 nm	NR	NR	NR	Cardiac dysfunction and increased collagen deposition. 16S rRNA/SCFA analysis: colon damage, microbiota disruption, decreased propionate → impaired FBP1 expression → cardiac injury (gut-heart axis).
Bu et al., 2025	Rats (intranasal)	PS nanoplastics	Nano-scale	NR	Intranasal	NR	Emphysema development. Mechanism: PS-NPs → macrophage SIGMAR1 binding → zDHHC14 release → SLC31A1 palmitoylation/stabilization → cuproptosis → TNF-α↑ → alveolar epithelial NLRP3/MMP-9 → pyroptosis → matrix degradation.
Zhang et al., 2025a	Pregnant mice (GD3.5–13.5)	PS-MPs (photo-aged vs. pristine)	1 μm	NR	Oral	GD3.5–13.5	Photo-aged PS-MPs caused fetal growth restriction and structural damage to placental labyrinthine chorionic layer. Greater toxicity than pristine form, associated with surface oxidation.
Cheng et al., 2025	Mice	Micro- and nanoplastics (MNPs)	NR	NR	NR	Gestational	Increased embryo resorption; decreased embryo weight, placental diameter and weight. Disrupted progesterone and estradiol production. Reduced spongiotrophoblast and labyrinth layer areas.
Chen et al., 2025	7-week-old male C57BL/6 mice	PS nanoplastics	Nano-scale	12.5 mg/kg	NR	NR	Brain: Cu accumulation → DLAT oligomerization, FDX1/LIAS/HSP70 dysfunction → cuproptosis; neuron loss, Nissl body reduction, synaptic plasticity impairment, learning/memory decline. GSH/SOD/Nrf2↓, ERK-MAPK activation. NAC reversed effects.
Yang et al., 2025	ApoE⁻/⁻ mice	PS microplastics	5 μm	1 μg/mL, 10 μg/mL	Oral	12 weeks	Body weight gain, dyslipidemia, aortic atherosclerotic plaque progression. BMP pathway activation, EndMT markers elevated in tissue. HUVECs confirmed oxidative stress and phenotypic changes in vitro.
Liu et al., 2025	Mice (chronic exposure)	PVC nanoplastics	Nano-scale	NR	NR	Chronic	ECG: cardiac electrical activity disruption; reduced EF and FS (ventricular dysfunction). Cardiac tissue: lysosomal autophagic flux impairment and mitochondrial dysfunction confirmed by ultrastructural and biochemical analysis.
Park et al., 2025	Mice	Microplastics	NR	NR	NR	NR	Hippocampus and cortex: DNA fragmentation, elevated neuroinflammation markers, increased kynurenine (KYN), 3-hydroxykynurenine, and quinolinic acid → kynurenine pathway activation → neuroinflammation and brain dysfunction.
Zheng et al., 2025	Mice (oral, 28-day chronic)	PS nanoplastics	Nano-scale	NR	Oral	28 days (chronic)	Retinal NP accumulation within 2 h of oral dosing; increased at 4 h. Chronic exposure: iBRB disruption, tight junction weakening, Nrf2/HO-1 suppression, ROS↑, Cleaved caspase-3↑ → retinal apoptosis.
Xiong et al., 2025b	Mice	PS nanoplastics	Nano-scale	30, 60, 100 mg/L	NR	42 days	Ventricular dilation, wall thinning, decreased ejection fraction, reduced heart rate and blood pressure. Dose-dependent cardiac structural and functional impairment.
Hong et al., 2024	Mice	PS microplastics	NR	10 mg/L	Drinking water	6 months (chronic)	Chronic kidney damage: inflammation, fibrosis. Ferroptosis induction; TGF-β1 release; fibroblast activation → renal fibrosis pathway.

Reference	Animal Model / Species	Polymer	Particle Size	Dose	Route of Exposure	Duration	Key Findings
Yin et al., 2024	Tumor-bearing mice (CAR-T cell model)	Microplastics (unspecified)	NR	NR	NR	NR	MP-exposed CAR-T cells showed reduced tumor suppression capacity. Tumor volume measurements confirmed attenuated anti-cancer efficacy. Rapamycin partially restored effects (mTORC1 pathway).
Fournier et al., 2020	Pregnant Sprague Dawley rats	Nanopolystyrene	20 nm	2.64×10^{14} particles	Intratracheal	24 h (acute)	NPs detected in maternal lung, heart, spleen, placenta, and fetal liver, lung, heart, kidney, brain. Isolated placental perfusion confirmed translocation. Maternal and fetal body weight: −7% and −8%, respectively.
Shen et al., 2022	Male C57BL/6 mice	PS microplastics	0.1 µm	1 mg/L	Drinking water	60 days	Liver: DNA damage, inflammatory response, fibrosis. cGAS/STING pathway activation; STING inhibitor reduced fibrosis and pro-inflammatory signaling.
Yin et al., 2023	Chickens (Gallus gallus)	PS microplastics	NR	Various doses	Oral	NR	Gut microbiota disruption → apoptosis-to-necrosis/pyroptosis transition → barrier weakening → LPS accumulation → liver immune and lipid metabolism dysregulation, mitochondrial imbalance, oxidative/ER stress. Caffeic acid and melanin metabolites identified as potential protectants.
Zhang et al., 2022 (human/environmental)	Humans (cross-sectional, n=40, Chengdu)	Mixed polymers (PU, Si, EVA, PE dominant)	Environmental MPs	High vs. low exposure area	Environmental (soil, air, intestinal fluid)	Cross-sectional	High-exposure group: significantly higher environmental and intestinal MP load. Nasal: ↑Klebsiella, Helicobacter; intestinal: ↑Bifidobacterium, Streptococcus, Sphingomonas. Beneficial bacteria (Bacteroides, Ruminococcus group, Dorea) decreased. Disrupted nasal-gut microbial symbiosis.
Wang et al., 2025 (eye)	Mice (topical)	PE	NR	Topical application	Topical (ocular)	NR	Dry eye signs, goblet cell loss, conjunctival inflammation.
Fusco et al., 2025	Mice (intravenous)	PS–Pd nanoplastics	50–200 nm	NR	Intravenous	NR	CyTOF tracking: rapid accumulation in liver, spleen, blood — selective in macrophages, monocytes, dendritic cells; low uptake in hepatocytes and lymphocytes. Selective mononuclear phagocytic system retention.
Kim et al., 2022	BALB/c nude mice + NCI-N87 gastric cancer cells	PS microplastics	Micro-scale	1.72×10^{4} particles/mL	Oral; intragastric	4 weeks (repeat)	PS accumulated in gastric tissue. NCI-N87: 2.9× increased migration, E-cadherin↓, N-cadherin↑, CD44↑, multi-drug resistance to bortezomib/paclitaxel/gefitinib/lapatinib/trastuzumab. ASGR2↑; silencing reversed effects. PS exposure may promote gastric cancer progression and therapy resistance.
Garcia et al., 2024	C57BL/6 mice (8–12 weeks, male and female)	PS or PS+PE+PLGA mixture	5 µm (PS); 1–4 µm (PE); 5 µm (PLGA)	0, 2, or 4 mg/week	Oral gavage	4 weeks (2×/week)	PS microspheres confirmed in brain, liver, kidney. Dose- and polymer-type-dependent metabolite profile changes in colon, liver, and brain. Intestinal barrier crossed; systemic organ distribution documented.
Wang et al., 2024a	Rats (oral gavage, 28 days)	PS nanoplastics	100 nm	Low (human min) and high (human max) dose	Oral gavage	28 days	Corpus cavernosum accumulation. High dose: erectile dysfunction, fibrosis, endothelial dysfunction, testosterone↓, oxidative stress↑, apoptosis↑. Low dose: accumulation without dysfunction.
Qu et al., 2024	BALB/c mice	PS microplastics	50 µm	NR	NR	NR	Leydig cell PS-MP internalization; histomorphological and ultrastructural damage; GnRH, FSH, LH, testosterone↓. GPX1 ubiquitin-mediated degradation via miR-425-3p → ER stress (PERK–eIF2α–ATF4–CHOP) → SRD5A2 promoter binding → testosterone metabolism ↑→ circulating level↓. HPT axis disruption. Anti-androgenic effects.
Merrill et al., 2023	Marine mammals (12 species, 22 individuals)	PE (most common) + mixed polymers	24.4–1,387 µm	Environmental (tissue analysis)	Oral (environmental)	Decades (archival samples)	MPs detected in acoustic fat pad, blubber, lung, melon. 68% of individuals: ≥1 tissue positive. PE most common; fiber most common shape. Concentrations: 0.59–25.20 µg/g, 0.04–0.39 particles/g. Evidence of translocation from oral route to fat-rich tissues.
Lee et al., 2023	ICR mice (single oral dose)	PP microplastics	~5 µm; 10–50 µm	Up to 2000 mg/kg	Oral (single dose)	24 h (IVIS); GI clearance	No toxicological adverse effects at doses up to 2000 mg/kg. NOAEL ≥ 2000 mg/kg. Cy5.5-labeled PP distributed mainly in GI tract, excreted within 24 h via feces.

Reference	Animal Model / Species	Polymer	Particle Size	Dose	Route of Exposure	Duratio n	Key Findings
Wang et al., 2024b	Male C57BL/6 mice (puberty onset)	PS microplastics	5 µm, 50 µm	100 µg/L, 1000 µg/L	Drinking water	10 weeks	50 µm particles: reduced colon mucus production, gut microbiota changes, decreased medial prefrontal cortex oxytocin, social behavior deficits, blood-brain barrier damage. Vagal pathway blockade improved neurobehavioral outcomes (gut-brain axis via oxytocin).
He et al., 2024	C57BL/6 mice	PS nanoplastics	100 nm	10 mg/L	Drinking water	3 months	PS-NPs crossed blood-retina barrier and accumulated in retina; increased oxidative stress; decreased scotopic ERG responses. Long-term: exacerbated light-induced photoreceptor degeneration and retinal inflammation. AMD-like transcriptomic profile.
Lee et al., 2024	Female C57BL/6 mice	PS microplastics	0.5 µm, 5 µm	0.3 mg (Nile Red labeled)	Oral gavage (2×/week)	12 weeks	Only 0.5 µm PS-MP accumulated in liver. Oxidative stress↑ (SOD, HNE-MA), IL-6↑, glucose/TG/ALT/AST↑, lipid droplets↑, liver weight index↑. 5 µm group: limited biochemical response. Sub-chronic submicron MP exposure → NASH-like hepatotoxicity.
Zhang et al., 2024 (MRI)	Mice (macrophage uptake)	Fe_3O_4@PS nanoplastics	Nano-scale	10 µg/mL (in vitro)	In vitro (macrophage)	NR	MRI signal-concentration standard curve established. Macrophage NP uptake: ICP 63.0%, MRI 57.7%. MRI validated as quantitative method for intracellular nanoplastic measurement.
Zhang et al., 2024 (kidney+Cd)	Male mice (oral gavage, 25 days)	PS nanoplastics + Cd	100 nm, 1 µm	NR	Oral gavage	25 days	100 nm PS: more severe oxidative damage and apoptosis than 1 µm. Cd+PS (both sizes): greater kidney damage than single exposures. Highest damage in Cd+100 nm+1 µm combination. Mitochondrial apoptotic pathway (Bax/Bcl2, p53, caspase-3).
Xiong et al., 2024	Female ICR-CD1 mice	PS nanoplastics + 3-BHA	Nano-scale	NR	Oral	35 days	↓Body weight, ovary/uterus indices; ↑histopathological damage, apoptosis, inflammation; ↓FSH and LH; ↑CAT and GSH-Px. FSTL1-mediated BMP4/TGF-β/SMAD pathway activation → female reproductive toxicity.
Liu et al., 2024 (lipidomics)	Mice (oral gavage, 14 days)	PS nanoplastics	Nano-scale	NR	Oral gavage	14 days	No overt intestinal histological damage. Lipidomics: 76 lipid species↑, 29↓; phosphatidylserine (9) and phosphatidylinositol (9) upregulated. Autophagic lipolysis disruption; PI3K signaling dysregulation; ceramide and cholesterol derivative changes.
He et al., 2023	Male Swiss mice	PS nanoplastics (pristine and UV-aged)	100 nm (aPS)	50 mg/kg/day	Oral gavage	7 days	Both groups: ↑serum glucose, dyslipidemia; ↑PI3K/p-AKT/GLUT4, SREBP-1, PPARγ, ATGL expression. UV-aged PS more potent effects on glucose metabolism and key protein expression. Self-regulatory metabolic compensation observed.
Guimarães et al., 2023	Male Swiss mice	PS nanoplastics	23 nm	6.5, 6500 ng/kg	NR	20 days	Anxiolytic-like behavior, altered anti-predator defense. Reduced brain DPPH radical scavenging and total glutathione. NP accumulation, erythrocyte DNA damage. Integrated biomarker response index; PCA/hierarchical clustering: similar response profiles at both doses.
Chen et al., 2024 (ducks)	Female Muscovy ducks	PVC microplastics + Cd	NR	NR	NR	2 months	Liver: cell ultrastructure disruption (nuclear/mitochondrial damage), reduced liver coefficient, elevated ALT. Oxidative stress-induced PCK1 deficiency → PI3K/AKT activation → hepatocyte lipid accumulation, fibrosis, glycogen deposition → apoptosis.
Sun et al., 2022 (epoxiconazole)	Mice	PS microplastics + epoxiconazole	5 µm	0.012 or 0.120 mg/kg (PS)	Oral	6 weeks	Combined exposure: more severe liver/kidney damage, oxidative stress, metabolic disorders than single exposures. Epoxiconazole disrupted gut microbiota → damaged intestinal barrier → facilitated PS bioaccumulation → impaired liver metabolism of epoxiconazole. Synergistic toxicity.
Liu et al., 2024 (zebrafish BMP)	Zebrafish embryos	PS nanoplastics	Nano-scale	1, 10, 100 µg/mL	Immersion	7 days	Reduced body length and hatching rate, skeletal deformities. sp7, sparc, smad1↑; runx2a, bmp2a, bmp4↓. ROS↑, sod1/cat↓, bcl-2/bax↑, Caspase-3↑. AKP activity inhibited.

Reference	Animal Model / Species	Polymer	Particle Size	Dose	Route of Exposure	Duration	Key Findings
							Multi-pathway skeletal toxicity via BMP pathway.
Huang et al., 2024 (CKD rat)	Juvenile Sprague Dawley rats (adenine CKD model)	Microplastics	NR	10 mg/L	Oral	3 weeks	MPs worsened CKD-related hypertension (systolic BP 155→140 mmHg with RBE treatment). RBE (resveratrol butyrate esters, 25 mg/L) normalized NO deficiency, modulated RAS system, gut microbiota, butyric acid production, and renal GPR41 expression. Antihypertensive mechanistic insight.
Saleh et al., 2025	C57BL/6 mice + 15-day recovery	PE microplastics	NR	6, 60, 600 μg/mL	Oral	15 days + 15-day recovery	Ileum: nuclear pyknosis, villus deformation/shortening, lamina propria degeneration, goblet cell hyperplasia/hypersecretion; P53 and Ki-67↑ at high dose (600 μg/day); intracellular MP accumulation. Recovery period reversed most changes.
Lopez et al., 2025	C57BL/6 mice (VSV and LCMV infection models)	PS nanoparticles	100 nm, 500 nm	NR	Oral	28 days	No significant impact on total or specific antibody titers (VSV), neutralizing capacity, or T cell phenotype/activation/exhaustion/function (LCMV). Short-term oral NP exposure does not impair adaptive antiviral immunity.
Lu et al., 2016 (zebrafish liver)	Zebrafish (7 days)	PS microplastics	5 μm, 20 μm	NR	Immersion	7 days	5 μm: accumulated in gill, liver, intestine; 20 μm: gill and intestine only. Both sizes: liver inflammation and fat accumulation; SOD and CAT↑ (oxidative stress). Metabolomics: altered lipid and energy metabolism profiles.
Wang et al., 2021 (mice)	Male C57BL/6 mice	PS microplastics	5 μm, 20 μm	0.1 mg/day	Oral	Chronic	Kidney: PS-MP accumulation, histopathological lesions, ER stress, inflammation and autophagy markers↑. Mitochondrial dysfunction, ER stress, inflammation, and autophagy identified as potential renal risk mechanisms.
Deng et al., 2017	Mice	PS microplastics	5 μm, 20 μm	NR	Oral	NR	Size-dependent tissue accumulation in liver, kidney, gut. Energy and lipid metabolism dysregulation, oxidative stress↑, serum neurotoxicity biomarker changes. First evidence of mammalian tissue accumulation with multi-biomarker adverse effects.
Kim et al., 2025 (PET)	ICR (CD-1®) mice	PET microplastics	<10 μm	NR	Oral	NR	Lung dominant accumulation (IVIS CT). Granulomatous inflammation at medium-high doses (dose-dependent). NOAEL: 1.75 mg/kg (males), 7 mg/kg (females). Small PET-MPs can induce persistent pulmonary inflammation.
Sun et al., 2023 (DEHP+PS)	C57BL/6 mice	PS microplastics + DEHP	5 μm	NR	Oral	NR	Co-exposure: strongest renal ROS induction; AMPK/ULK1 and Ppargc1α/Mfn2↑; LC3/Beclin-1↑; autophagosome accumulation. DEHP+PS synergistically activated AMPK/ULK1 → renal autophagy and nephrotoxicity.
Xiong et al., 2023 (kidney RNA-seq)	C57BL/6 mice (long-term)	PS microplastics	80 nm, 0.5 μm, 5 μm	NR	NR	Long-term	All 3 sizes: progressive renal tissue damage, inflammation, oxidative stress, apoptosis → kidney injury and fibrosis. Transcriptomics: 80 nm altered immune response genes; 0.5 μm and 5 μm altered circadian rhythm genes.
Meng et al., 2022	Chickens	PS microplastics	NR	1, 10, 100 mg/L	Drinking water	6 weeks	Kidney: mitochondrial dynamic disruption (MFN1/2, OPA1, DRP1↑), structural damage, ↓SOD/CAT/GSH/T-AOC (oxidative stress), NF-κB P65/TNF-α/iNOS/IL-1β/IL-6↑ (inflammation), RIP1/RIP3/MLKL necroptosis pathway activated → concentration-dependent cell death.
Chen et al., 2022 (PS+Cd, kidney)	Male mice (oral gavage, 25 days) + female ICR mice (35 days)	PS nanoplastics + Cd; PS nanoplastics + 3-BHA	NR (PS-NP) / nano-scale	NR	Oral gavage	25 days / 35 days	PS+Cd (male): 100 nm > 1 μm kidney damage; combined > single; Bax/Bcl2, p53, caspase-3 pathway. PS+3-BHA (female): ↓ovary/uterus indices; FSTL1/BMP4/TGF-β/SMAD pathway; reproductive toxicity.

Abbreviations: PS = polystyrene; PE = polyethylene; PP = polypropylene; PVC = polyvinyl chloride; PET = polyethylene terephthalate; PLGA = poly(lactic-co-glycolic acid); DEHP = di(2-ethylhexyl) phthalate; Cd = cadmium; Mn = manganese; 3-BHA = 3-butylhydroxyanisole; NR = not reported; MP = microplastic; NP = nanoplastic; ROS = reactive oxygen species; SOD = superoxide dismutase; CAT = catalase; GSH = glutathione; ER = endoplasmic reticulum; NF-κB = nuclear factor kappa B; IL = interleukin; TNF-α = tumor necrosis factor alpha; FBP1 = fructose-1,6-bisphosphatase 1; NET = neutrophil extracellular trap; EndMT = endothelial-mesenchymal transition; BMP = bone

morphogenetic protein; PPARγ = peroxisome proliferator-activated receptor gamma; SCFA = short-chain fatty acids; iBRB = inner blood-retina barrier; NOAEL = no-observed-adverse-effect level; GD = gestational day; HPT = hypothalamic-pituitary-testis; CKD = chronic kidney disease; NASH = non-alcoholic steatohepatitis; AMD = age-related macular degeneration; CyTOF = cytometry by time-of-flight.

Table S3. Summary of Selected Human Studies on Microplastic/Nanoplastic Exposure and Detection

Reference	Study Design / Population	Sample / Tissue	n	Predominant Polymer(s)	MP Size Range	Key Findings
Schwabl et al., 2019	Prospective case series; healthy volunteers	Feces	8	PP, PET (most common among 9 types)	50–500 µm	All samples contained MPs; mean 20 particles/10 g feces. First study confirming involuntary MP ingestion in healthy adults via stool analysis.
Ibrahim et al., 2020	Surgical patients (colectomy)	Colon tissue	11	PC (90%), PA (50%), PP (40%) among tested filaments	28.1 ± 15.4 particles/g tissue; fiber/filament form 96.1%	MPs detected in all colon samples (mean 331 particles/person). 96.1% filament form; 73.1% transparent. PC, PA, PP most common. MPs widely distributed in human colon.
Braun et al., 2021	Clinical setting; cesarean deliveries	Placenta, meconium	2 cases	PE, PP, PS, PU (PU also found in OR air — potential contamination)	>50 µm	First clinical detection of MPs in human placenta and meconium. PU likely from OR air contamination. Highlights need for nano-scale MP investigation.
Ragusa et al., 2022	Prospective pilot; postpartum women	Breast milk	34	PE, PVC, PP (most common)	2–12 µm	MPs detected in 26/34 samples. No significant association with age, cosmetic use, seafood/packaged food consumption. First documentation of MP contamination in human breast milk.
Jenner et al., 2022	Tissue analysis (lung autopsy)	Lung tissue	13	PP (23%), PET (18%), resin (15%); 12 polymer types	Mean 1.42 ± 1.50 particles/g (background-corrected: 0.69 ± 0.84)	MPs in 11/13 samples. Lower lobe highest concentration (3.12 ± 1.30 particles/g). Confirms inhalation as significant exposure route with regional distribution differences.
Leslie et al., 2022	Biomonitoring; healthy volunteers	Whole blood	22	PET, PE, styrene polymers (PS/EPS/ABS); PMMA, PP below LOD	≥700 nm	MPs detected in blood from majority of donors; mean total 1.6 µg/mL. First quantitative biomonitoring confirming plastic particles enter human circulation.
Yan et al., 2022	Case-control; IBD patients vs. healthy controls	Feces	IBD and healthy (NR)	PET (22.3–34.0%), polyamide (8.9–12.4%); 15 types	Sheet and fiber forms dominant	IBD patients: 41.8 particles/g dry weight vs. 28.0 in controls. MP level positively correlated with IBD severity. Plastic packaging and dust identified as likely sources.
Horvatits et al., 2022	Case series; liver cirrhosis patients vs. controls	Liver, kidney, spleen	6 cirrhosis + 5 controls	6 polymer types (4–30 µm)	4–30 µm	MPs in all cirrhotic tissue samples; none in controls. Tissue-specific accumulation of MPs in cirrhotic liver. Suggests disease state may facilitate or reflect greater MP deposition.
Wu et al., 2023	Retrospective case series; cardiovascular surgery	Thrombus samples	26	Phthalocyanine (21), Hostasol-Green (1), LDPE (1); iron compounds and metal oxides	2.1–26.0 µm	MPs detected in 16/26 thrombus samples (median 5 particles/sample). Positive correlation between thrombus particle count and platelet levels ($p<0.01$). First Raman-based documentation of microparticles in human thrombi.
Guan et al., 2023	Prospective observational; patients with various conditions	CSF, whole blood, cyst fluids, effusions (n=104 patients)	104	PS, PP, PA-6, PET + others; 78–83% fiber form	20–100 µm (most); some <20 µm	702 microparticles detected across body fluids. Highest density in effusions (~80/sample); lowest in CSF. Strict PDQC protocol applied. Widespread systemic and tissue-specific MP accumulation confirmed.
Zhao et al., 2023	Cross-sectional; male reproductive health	Testis (6), semen (30)	36 men	Testis: PS (67.7%); Semen: PE, PVC dominant	21.76–286.71 µm; 67–80.6% in 20–100 µm range	Semen: mean 0.23 ± 0.45 particles/mL; testis: 11.60 ± 15.52 particles/g. Fragment form in testis; fiber and film also in semen. First study documenting MP contamination in human male reproductive system.
Yang et al., 2023 (cardiac)	Prospective; cardiac surgery patients	Pericardium, epicardial/pericardial fat, myocardium, LAA; blood (pre-/post-op)	15	9 MP types including PMMA (organ-specific); max 469 µm in tissue; 184 µm in blood	Up to 469 µm	MPs in all cardiac tissue types and blood. Post-operative blood sample showed type/size distribution changes. PMMA in LAA, epicardial/pericardial fat not attributable to surgical contamination. First confirmation in fully enclosed cardiac structures.

Reference	Study Design / Population	Sample / Tissue	n	Predominant Polymer(s)	MP Size Range	Key Findings
Zhu et al., 2023 (placenta)	Tissue analysis; human placentas	Placenta	17	PVC (43.27%), PP (14.55%), PBS (10.90%); 11 polymer types	20.34–307.29 μm; 80.29% <100 μm	Mean 2.70 ± 2.65 particles/g (0.28–9.55). PVC and PP mostly <200 μm; larger particles fiber form. Baseline data for fetal exposure risk assessment.
Abbasi & Turner, 2021	Cross-sectional exposure study; Iranian adults	Skin (hand/face), hair, saliva	>2,000 adults	PE-PET, PP (most common), fiber form	<100 μm dominant	Total >16,000 MPs detected. Highest in hair (>7,000 total; mean 3.5/person/day), lowest in saliva (~650; 0.33/person/day). Males ~2× higher exposure. High variability in 7-day sampling. Urban vs. rural: similar counts except arid/remote areas lower.
Huang et al., 2022 (sputum)	Retrospective case series; respiratory patients	Sputum	22	PU (dominant), polyester, chlorinated PE, alkyd varnish; 21 types total	Median 75.43 μm (IQR: 44.67–210.64 μm)	MPs in all sputum samples. Smoking and invasive procedure history significantly associated with MP quantities ($p<0.05$). Confirms inhalation-ingestion route for respiratory patients.
Li et al., 2024 (semen)	Prospective; men attending pre-marital health check	Semen	40	PS (31%), 8 polymer types	0.72–7.02 μm	Mean 2 MPs/sample. PS group: higher progressive sperm motility vs. PVC group (43.52% vs. 19.04%). Sperm morphological abnormalities without polymer-specific significance. Non-occupationally exposed male population.
Nihart et al., 2025 (brain)	Decedent tissue analysis (autopsy)	Brain, kidney, liver	Multiple donors	PE (dominant, especially brain); nano-scale shard-like particles	Nanoscale (NM scale)	Brain had highest MNP concentration vs. kidney and liver. Brain PE ratio higher than other organs. 2016 vs. 2024 samples: significant increase in liver and brain ($p=0.01$). Dementia donors: higher brain MNP accumulation. Particles found in vessel walls and immune cells.
Zhu et al., 2024 (multi-organ)	Tissue analysis (surgical/autopsy)	Lung, small intestine, large intestine, tonsil	NR	PVC (dominant in all tissues); >20 μm range	>20 μm; most common 20–100 μm	Highest in lung (14.19 ± 14.57 particles/g). PVC most common across all tissues. Women significantly higher MP abundance than men ($p<0.05$). PVC flagged for high polymer hazard index.
Zhang et al., 2024 (multi-site, sperm)	Multi-site study; male participants in China	Semen, urine	113	PS, PP, PE, PTFE	Micro-scale	MPs detected in semen and urine. PTFE exposure significantly associated with reduced sperm count, concentration, and motility. Each additional MP type associated with decreased semen quality. Urine analysis proposed as potential early biomarker.
Özsoy et al., 2024 (stomach)	Forensic autopsy cases	Stomach contents	26 cadavers	Mixed (fiber 52%, fragment 40%, film 8%)	NR; daily intake estimated at 32.2 particles	All samples contained MPs; mean 9.4 ± 10.4 particles/person. Lower than fecal estimates; confirms gastric MP presence in humans.
Yang et al., 2024 (joints)	Cross-sectional; joint arthroplasty patients	Synovium (hip and knee)	45	9 types; most common PS, PP, EVA	1.16–10.77 particles/g; range 25–407 μm	Mean 5.24 ± 2.07 particles/g. Hip > knee. Transcriptomics: 3× MP abundance → heat shock protein response amplification. Potential link between joint MP accumulation and local cellular stress.
Lee et al., 2024 (blood coagulation)	Prospective cross-sectional; healthy adults	Whole blood	36	PS, PP (most common)	NR; μ-FTIR (≥ detection limit)	MPs in 88.9% of participants; mean 4.2 MPs/mL. Plastic food container use correlated with higher blood MP levels. High MP load (≥3 MPs/mL): elevated aPTT, CRP, fibrinogen → coagulation and cardiovascular risk implications.
Sun et al., 2024 (endometrium)	Cross-sectional; women undergoing endometrial evaluation	Endometrium	NR	Ethylene-acrylic acid copolymer (small MPs), PE (large MPs); 13 types; 6 high-abundance types	88.35% in 20–100 μm range; median 21 particles/100 mg	0–117 particles/100 mg (median 21). No age/BMI correlation. Drinking habits and gum-chewing associated with higher MP exposure. First documentation of MPs in human endometrium.
Yun et al., 2024 (placenta Raman)	Case series; postpartum placenta analysis	Placenta	50 postpartum women	PTFE (most common), PS, ABS; 7 polymer types	Mean 2.35 ± 1.25 μm (1.03–6.84 μm)	40 MPs in 31/50 placentas. No significant differences in maternal age, gestational age, newborn size/weight between PTFE/PS-positive and controls. Short-term clinical outcomes unaffected.
Tuna et al., 2023	Prospective; allergic rhinitis patients vs. healthy controls	Nasal lavage	36 allergic rhinitis + 30 controls	NR	NR	MPs detected in all participants. MP density significantly higher in allergic rhinitis group ($p=0.027$). Methodological concerns regarding contamination controls noted.

Reference	Study Design / Population	Sample / Tissue	n	Predominant Polymer(s)	MP Size Range	Key Findings
Zhang et al., 2024 (nasal lavage/masks)	Prospective; university students with different mask types	Nasal lavage	113	PP (NE group 58.70%), PC (SM: 49.49%, CM: 54.29%)	Various	Surgical mask (SM) group: highest MP abundance (41.24 ± 1.73 particles/g). Natural exposure (NE) and cotton mask (CM) groups significantly lower ($p<0.01$). Longer mask use → higher nasal MP levels ($\beta=0.28$, $p<0.05$). Mask use influences respiratory MP exposure.
Yang et al., 2025 (skeletal)	Human tissue analysis + in vivo mouse model	Bone, cartilage, intervertebral disc	24 samples	PP (35%), EVA (30%), PS (20%)	25–407 μm; disc highest (159.5 μm avg)	Disc highest (61.1 ± 44.2 particles/g) > bone (22.9) > cartilage (26.4). 4-week in vivo exposure: blood-borne translocation confirmed; TNF-α, PINP, TRACP-5b↑ → inflammatory and bone morphogenetic cytokine changes.
He et al., 2025 (Alzheimer's CSF)	Biomarker case-control study; amyloid-positive vs. negative individuals	Cerebrospinal fluid (CSF)	Cohort 1: 17 amyloid+, 15 amyloid−; Cohort 2: 11 amyloid+	PP, PVC, PE, PS	NR	Amyloid-positive: significantly higher PE and PVC in CSF vs. amyloid-negative. PE negatively correlated with CSF Aβ42 and MMSE; positively with MMSE decline rate. CSF PE and PVC: AUC>0.8 for discriminating amyloid status. MPs may be linked to cognitive decline in Alzheimer's disease.
Pan et al., 2025 (colorectal cancer)	Tissue analysis; CRC patients	Peritumoral and tumor tissue	NR	PVC, PE (primary)	NR (SEM and LDIR)	MPs detected in both peritumoral and tumor tissue. Greater diversity and distribution of MPs in tumor regions. Clathrin-α overexpression in CRC samples → facilitates MP cellular uptake. Potential link between MP exposure and CRC pathogenesis.
Wang et al., 2025 (pregnancy liver enzymes)	Prospective cohort	Placenta; umbilical cord blood (liver enzymes)	1,057	PVC, PP, PBS (dominant; ~89% positive)	Median 12 particles/10 g tissue	High PVC: ↑ALP ($\beta=28.07$; $p=0.01$). PP: ↑ALT ($\beta=0.63$; $p=0.05$) and AST ($\beta=3.42$; $p=0.01$). PP+total MP: ↑GGT ($p<0.01$). BKMR/g-comp mixed exposure analysis confirmed ALP, AST, GGT elevations. Prenatal MP exposure may affect fetal hepatic function.
Jochum et al., 2025 (preterm birth)	Case-control; preterm vs. term birth placentas	Placenta	71 PTB + 87 normal	PVC, PET, PU, PC (significantly elevated in PTB group)	Micro/nano scale (Py-GC/MS)	PTB placentas: 28% higher total MNP (224.7 vs. 175.5 μg/g; $p=0.038$). PVC and PC: independent predictors of preterm birth (logistic regression). PET, PU, PC: inverse correlation with gestational age and birth weight.
Song et al., 2024 (urine)	Cross-sectional; urban vs. rural adults (Chongqing)	Urine	12 volunteers	Py-GC/MS: PE dominant; LDIR: ACR dominant; also PVC, PA66, PMMA, PU, PP, PET	Py-GC/MS: 1.50 mg/kg avg; LDIR: 15.17 particles/kg avg	Urban samples: higher quantity and polymer diversity than rural. Plastic toy contact and personal care product use correlated with MNP exposure. First study combining Py-GC/MS and LDIR simultaneously for urine MNP analysis.
Salvia et al., 2023	Method development; healthy individuals	Peripheral blood	NR	NR (Nile Red-stained NPs, 1–1000 nm)	1–1000 nm	Flow cytometry/nanocytometry method validated for detecting nanoplastics in human peripheral blood. Reliable environmental exposure marker for NP detection without processing artifacts.
Erdely et al., 2025 (nanoplastics)	Morphological/chemical characterization; deceased donors	Brain, kidney, liver	5 donors	Nano-scale fibers (78–83% elongated fiber morphology)	Brain: 171.2 nm; Kidney: 124.4 nm; Liver: 147.6 nm (mean lengths)	Organ-specific nanoplastic size profiles. Brain consistently largest particles. Inter-donor tissue differences greater than intra-donor differences. Systemic circulation → organ-specific accumulation patterns.
Massardo et al., 2024	Prospective observational pilot; kidney donors and healthy subjects	Kidney tissue, urine	NR	PE, PS (most frequent); hematite, Cu-phthalocyanine pigments also identified	Kidney: 1–29 μm; Urine: 3–13 μm	26 MPs total across urine and tissue samples. First microRaman spectroscopy study detecting MPs in human urine and kidney tissue simultaneously.
Campen et al., 2024 (preprint)	Decedent tissue analysis (autopsy, Albuquerque)	Brain, liver, kidney (2016 and 2024 samples)	NR	PE (dominant, especially brain); 12 polymers quantified by Py-GC/MS	Nano-scale (TEM confirmed)	Brain higher MNP than liver and kidney. All organs: significant increase 2016→2024. Brain PE proportion higher than other organs. TEM: aged/fractured nano-scale particle morphology confirmed.
Refosco et al., 2025 (feces Norway)	Pilot cross-sectional; Norwegian adults (seafood vs. non-seafood consumers)	Feces	18	PP (72%), PE (44%), PS (16%)	12.5–4065 μm	132 MPs in 17/18 samples. No association between seafood consumption or other food groups and MP abundance. Standardized

Reference	Study Design / Population	Sample / Tissue	n	Predominant Polymer(s)	MP Size Range	Key Findings
						extraction protocols and regional individual variability needed.
Jahedi et al., 2025 (respiratory fluids)	Observational; Iranian respiratory patients	Urine, sputum, BAL fluid	30	Urine: PE, PP, PS; Sputum: PU dominant (broad diversity); BALF: urine-like polymer composition	Urine: small 20–100 µm; Sputum: wide range (358 MPs)	Urine: 9 MPs (small, green/red PE/PP/PS fibers). Sputum: 358 MPs (broadest size range and polymer diversity). BALF: 123 MPs (larger fibers/fragments). Physiological fractionation differences suggest distinct deposition and clearance mechanisms between body fluids.
Yang et al., 2024 (ACS/coronary)	Case-control; chest pain patients (19 controls, 82 ACS)	Blood	101	PE, PVC, PS, PP	NR (Py-GC/MS)	ACS patients, especially AMI group: significantly higher blood MP levels than controls. Higher MP in moderate-high vs. low CAD risk. MP levels positively correlated with IL-6, IL-12p70, B lymphocytes, NK cells. MPs may contribute to vascular and immunoinflammatory pathology in ACS.
Fusco et al., 2025 (blood/CyTOF)	Ex vivo human blood + in vivo mouse model	Human blood (ex vivo)	NR	PS-Pd nanoplastics (50–200 nm)	50–200 nm	Rapid and selective accumulation in macrophages, monocytes, and dendritic cells in blood and organs (liver, spleen). Low uptake in hepatocytes and lymphocytes. CyTOF single-cell mass cytometry enables precise immune cell-level NP tracking.
Zhang et al., 2022 (microbiota)	Cross-sectional observational; factory workers vs. park visitors	Soil, air, intestinal fluid, nasal/intestinal microbiota (16S rDNA)	40 (20 high, 20 low exposure)	PU, Si, EVA, PE (prominent in high exposure)	Environmental MPs	High-exposure: ↑Klebsiella, Helicobacter (nasal); ↑potentially pathogenic bacteria, ↓Bacteroides, Ruminococcus, Dorea, Fusobacterium, Coprococcus (intestinal). Disrupted nasal-gut symbiosis. Microplastic load correlates with microbiota dysbiosis in both upper and lower tracts.
Wang et al., 2025 (dry eye)	Case series; dry eye patients	Tear fluid, meibum	45	PE (most common); 5 MP types	NR (FTIR)	MPs in all samples. PE levels significantly correlated with Schirmer I test and FBUT values. First documentation of MPs in human tear fluid associated with dry eye disease severity markers.

Abbreviations: PS = polystyrene; PE = polyethylene; PP = polypropylene; PVC = polyvinyl chloride; PET = polyethylene terephthalate; PC = polycarbonate; PA = polyamide; PU = polyurethane; PMMA = polymethylmethacrylate; PBS = polybutylene succinate; EVA = ethylene-vinyl acetate copolymer; PTFE = polytetrafluoroethylene; LDPE = low-density polyethylene; ABS = acrylonitrile butadiene styrene; ACR = acrylate; NR = not reported; MP = microplastic; NP = nanoplastic; MNP = micro/nanoplastic; IBD = inflammatory bowel disease; CRC = colorectal cancer; ACS = acute coronary syndrome; AMI = acute myocardial infarction; CAD = coronary artery disease; CSF = cerebrospinal fluid; BALF = bronchoalveolar lavage fluid; BAL = bronchoalveolar lavage; LD-IR = laser direct infrared; FTIR = Fourier-transform infrared spectroscopy; Py-GC/MS = pyrolysis gas chromatography-mass spectrometry; μ-FTIR = micro-Fourier-transform infrared spectroscopy; LDIR = laser direct infrared imaging; SEM = scanning electron microscopy; Raman = Raman spectroscopy/microspectroscopy; aPTT = activated partial thromboplastin time; CRP = C-reactive protein; FBUT = fluorescein tear film break-up time; MMSE = Mini-Mental State Examination; LOD = limit of detection; ALP = alkaline phosphatase; ALT = alanine aminotransferase; AST = aspartate aminotransferase; GGT = gamma-glutamyl transferase; PTB = preterm birth; OR = operating room; GD = gestational day; BKMR = Bayesian kernel machine regression; AUC = area under the curve.
